# Acute kidney injury—how does automated detection perform?

**DOI:** 10.1093/ndt/gfv094

**Published:** 2015-04-28

**Authors:** Simon Sawhney, Nick Fluck, Angharad Marks, Gordon Prescott, William Simpson, Laurie Tomlinson, Corri Black

**Affiliations:** 1 Division of Applied Renal Research Collaboration, University of Aberdeen, Aberdeen, UK; 2 NHS Grampian, Aberdeen, UK; 3 London School of Hygiene and Tropical Medicine, London, UK

**Keywords:** acute kidney injury, diagnosis, epidemiology, screening

## Abstract

**Background:**

Early detection of acute kidney injury (AKI) is important for safe clinical practice. NHS England is implementing a nationwide automated AKI detection system based on changes in blood creatinine. Little has been reported on the similarities and differences of AKI patients detected by this algorithm and other definitions of AKI in the literature.

**Methods:**

We assessed the NHS England AKI algorithm and other definitions using routine biochemistry in our own health authority in Scotland in 2003 (adult population 438 332). Linked hospital episode codes (ICD-10) were used to identify patients where AKI was a major clinical diagnosis. We compared how well the algorithm detected this subset of AKI patients in comparison to other definitions of AKI. We also evaluated the potential ‘alert burden’ from using the NHS England algorithm in comparison to other AKI definitions.

**Results:**

Of 127 851 patients with at least one blood test in 2003, the NHS England AKI algorithm identified 5565 patients. The combined NHS England algorithm criteria detected 91.2% (87.6–94.0) of patients who had an ICD-10 AKI code and this was better than any individual AKI definition. Some of those not captured could be identified by algorithm modifications to identify AKI in retrospect after recovery, but this would not be practical in real-time. Any modifications also increased the number of alerted patients (2-fold in the most sensitive model).

**Conclusions:**

The NHS England AKI algorithm performs well as a diagnostic adjunct in clinical practice. In those without baseline data, AKI may only be seen in biochemistry in retrospect, therefore proactive clinical care remains essential. An alternative algorithm could increase the diagnostic sensitivity, but this would also produce a much greater burden of patient alerts.

## INTRODUCTION

Acute kidney injury (AKI) is a serious condition complicating one in seven hospital admissions [1]. It is usually diagnosed from rapidly deteriorating blood tests (serum creatinine) or urine output. It can occur in any clinical setting, leading to substantially increased hospital mortality (one in three in severe AKI) [2], morbidity and healthcare costs (£1billion/year in NHS England) [1, 2].

AKI must be recognized early with appropriate intervention or monitoring. In the UK, a National Confidential Enquiry into Patient Outcome and Death (NCEPOD) found suboptimal care in 50% of patient deaths from AKI, with delays in care and preventable harm [3]. A simple system is therefore needed to improve early detection in AKI across healthcare settings [4].

In response, NHS England plans a mandatory national automated algorithm for detecting AKI [5]. The algorithm is based on the Kidney Disease: Improving Global Outcomes (KDIGO) AKI definition [6]. Changes in serum creatinine are tracked in a biochemistry system with each new (index) test compared with previous (reference) results. An AKI ‘alert’ can be generated if sufficient change in creatinine has occurred in a short space of time. One of three criteria should be satisfied, differing in the time period of creatinine change (Table 1). AKI stage can also be calculated, based on the magnitude of creatinine rise [6]. This algorithm may help clinicians recognize AKI early, and may be of use in audit and clinical research [5].

**Table 1.** NHS automated AKI algorithm

AKI criteria	Definition for AKI (one of the three)
Criterion 1	Serum creatinine ≥1.5 times higher than the median of all creatinines 8–365 days ago
Criterion 2	Serum creatinine ≥1.5 times higher than the lowest creatinine within 7 days
Criterion 3	Serum creatinine >26 μmol/L higher than the lowest creatinine within 48 h
AKI Stage	Classification requirements
Stage 1	Rise in creatinine of >26 or index/reference ≥1.5 and <2
Stage 2	Index/reference ≥2 and <3
Stage 3	Index/reference ≥3 or ≥1.5 and index creatinine >354 μmol/L (or three times the upper reference interval if age <18)

AKI can be diagnosed if one of three criteria is met. Staging is based on a comparison of a serum creatinine (index) with a reference test. Where a creatinine is outside the reference range but a previous creatinine within 1 year is unavailable, the test is ‘flagged’ abnormal (with chronicity uncertain).

The NHS England AKI algorithm is novel, but an awareness of its strengths, weaknesses and any unintended consequences is essential. Previous automated AKI detection systems have been reported, but not on a national scale [7–12], and while their successful implementation is encouraging, there are calls for them to prove their diagnostic value [13]. In particular, it is necessary to recognize the acute changes of AKI from chronic kidney disease (CKD)—a challenge in routine healthcare when not all patients have recent tests for comparison [14]. The NHS England algorithm also differs from previous approaches for identifying AKI. Previous algorithms have varied in definitions and been of lower complexity using only a single reference criterion rather than a combination [7–11]. Some have also involved a human step in determining reference creatinine [7, 9]. In clinical research, a longer look-back period [14, 15], imputation of missing results [16, 17] or a subsequent fall in creatinine during follow-up have all been suggested when previous results are unavailable [2, 18, 19].

Once the algorithm identifies possible AKI, it is up to the clinician to interpret. Consequently, to maximize clinical benefit from an AKI algorithm, clinicians must understand which patients may be incorrectly identified (false positives), whether AKI patients may be missed (false negatives), and how to act when warned by an alert. Similarly, to study AKI prognosis it is important to first understand how different AKI definitions could cause selection biases in clinical research. An alternative standard is needed to compare these definitions.

Clinically coded AKI provides an alternative mechanism to identify a subset of AKI that has been confirmed clinically by the treating physicians and subsequently coded. World Health Organisation International Classification of Disease (ICD-10) codes for diagnosis are routinely collected on hospital discharge in the UK and are used for healthcare planning [20]. ICD-10 codes are highly specific for AKI, but only report a subset of all AKI [21]. The high specificity of ICD-10 AKI has been shown in US administrative coding (for billing) [22] and similar high specificity (95%) was observed in a single-centre UK study using ICD-10 codes in 2005 and 2010 [23]. Thus, while not all AKI patients are coded, ICD-10 AKI patients have a reliable diagnosis and should not be missed by a sensitive screening algorithm.

**FIGURE 1:**
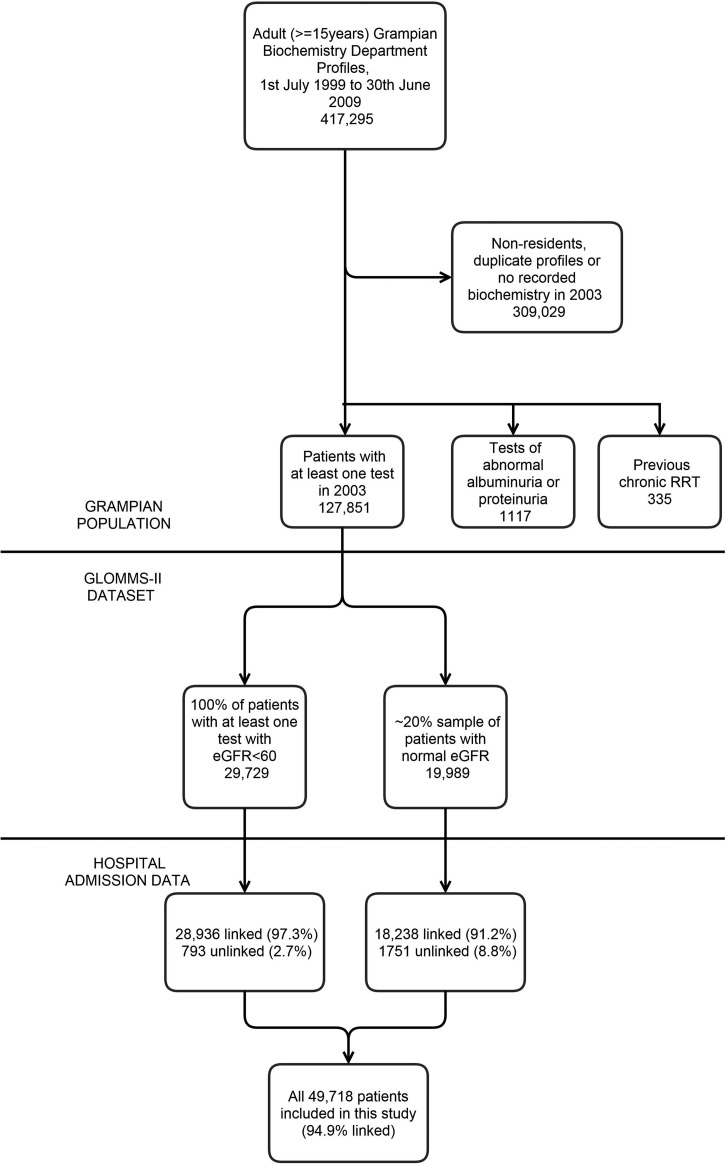
Summary of the GLOMMS-II Study Population. eGFR, estimated glomerular filtration rate; RRT, renal replacement therapy.

The Grampian Laboratory Outcomes, Morbidity and Mortality Study-II (GLOMMS-II) cohort is a population based cohort from 1 of 14 health authorities in NHS Scotland with an adult population of 438 332 [24]. It includes all patients from the index year (2003) with renal impairment (estimated glomerular filtration rate, eGFR < 60 mL/min/1.73m^2^) and a sample of patients with normal kidney function (eGFR ≥ 60 mL/min/1.73m^2^) (Figure 1). GLOMMS-II is an established platform for observing the prognosis of kidney diseases and contributes to the international CKD Prognosis Consortium [25]. This large regional cohort has been linked to ICD-10 data and provides an opportunity to rapidly contrast the implications of different biochemical definitions of AKI [26]. While cohort inception was 2003 and coding of AKI has since increased, the specificity of ICD-10 AKI codes has remained high through this period [23].

The NHS England algorithm is mandatory in England and is based on sound principles, but as yet is not validated. Therefore, we report here how their algorithm performs in our own health authority in NHS Scotland. We used GLOMMS-II to compare the NHS AKI algorithm and other recognized biochemistry definitions with coded ICD-10 AKI episodes. We studied how many patient-alerts these definitions involved and what proportion of ICD-10 AKI coded patients were detected (sensitivity).

## MATERIALS AND METHODS

### Study population—the GLOMMS-II cohort

Grampian is a region of north-east Scotland served by a single biochemistry service, which provided the biochemistry in GLOMMS-II. The service processes blood samples regardless of clinical setting, whether hospital, community or private (i.e. outside NHS). All serum creatinines were isotope dilution mass spectrometry aligned. The estimated mid-year resident adult (≥15 years) population of Grampian was 438 332 in 2003, of which 127 851 (29.2%) had at least one serum creatinine test. For linkage, we included all adults with an abnormal kidney blood test (eGFR < 60 mL/min/1.73m^2^) in 2003 (*n* = 29 729) and a 20% sample of those with normal tests (*n* = 19 989) as subgroups for study (Figure 1).

### Data linkage

We linked GLOMMS-II to the Scottish Renal Registry and local renal management system to exclude patients receiving chronic renal replacement therapy (RRT). We also linked GLOMMS-II to nationally collected hospital episode data including hospital diagnoses for each admission over 10 years (1999–2009) using ICD-10. Data linkage involved deterministic matching using the Community Health Index, a unique identifier for all residents in Scotland. In this study, 94.9% of patients could be linked to at least one hospital admission for retrieval of ICD-10 codes. We obtained approval from the appropriate Privacy Advisory and ethics panels. The data were hosted and managed by Grampian Data Safe Haven [27].

### Population characteristics

We collected morbidity data for ischaemic heart disease, cardiac failure, diabetes mellitus and previous renal disease from ICD-10 codes using a previously described look-back period of 5 years (1 January 1998–31 December 2002) [28]. We also collected data on patient location at AKI diagnosis, frequency of blood testing and baseline renal impairment. We used the four-variable Modification of Diet in Renal Disease (MDRD) eGFR equation to describe baseline renal impairment.

### Reference standard—ICD-10 AKI

We extracted all ICD-10 codes for acute renal failure (N17) in any diagnostic position (maximum six available) on discharge between 1 January 2003 and 31 December 2003. We labelled patients as ICD-10 AKI positive or negative based on whether they had at least one ICD-10 AKI episode in 2003.

### Biochemical AKI algorithm

For each patient, we identified the first episode of biochemical AKI in 2003. We used the NHS England AKI algorithm, requiring at least one of criteria 1–3 in Table 2. For instance, criterion 1 requires an index creatinine ≥1.5-fold higher than the median creatinine of the previous 8–365 days. Three other criteria, 4–6, based on methods used in previous studies were also assessed [2, 8, 9, 11, 18, 19]. We deviated from the algorithm in two respects. As sample time data were incomplete, we calculated creatinine changes using days rather than hours. Where blood tests were repeated on the same day, we included the highest creatinine. In both cases, we checked the impact on sensitivity analyses.

**Table 2.** AKI criteria based on the relationship between a blood test of interest and a reference creatinine

NHS algorithm	AKI criteria	Time period	Reference creatinine	Relationship
Y	Criterion 1	8–365 days	Median of all creatinines previous 8–365 days	Ratio ≥ 1.5
Y	Criterion 2	1–7 days	Lowest creatinine in previous 7 days	Ratio ≥ 1.5
Y	Criterion 3	48 hours	Lowest creatinine in previous 48 h	Rise > 26 μmol/L
N	Criterion 4	30 days (future)	Lowest creatinine within 30 days after index	Ratio ≥ 1.5
N	Criterion 5	3 years	Most recent creatinine in previous 3 years	Ratio ≥ 1.5
N	Criterion 6	8–365 days	Lowest creatinine in previous 8–365 days	Ratio ≥ 1.5

The NHS AKI algorithm required one of criteria 1–3 (denoted ‘Y’) to be met. Criteria 4–6 are alternative criteria used in previously in clinical research.

### Biochemical AKI stage

We calculated AKI stage based on creatinine rises in NHS England algorithm criteria (Table 1). We reported the highest stage within 30 days of first detection. If more than one reference creatinine was available the value providing the highest AKI stage was used. We also reported the stage at first AKI detection to assess progression to AKI Stage 3 from lower AKI stages.

### Analysis

We summarized patient characteristics at baseline including morbidities, number with biochemical AKI and ICD-10 AKI. We stratified by the biochemical AKI stage, comparing co-morbidities, ICD-10 AKI, number of alerts and patient location at first AKI detection. We also compared the characteristics of ICD-10 AKI patients detected and missed by the algorithm.

For biochemical AKI, we reported ‘sensitivity’ using ICD-10 AKI as the reference standard. We did this first for each biochemical criterion, then for the NHS England AKI algorithm (criteria 1–3, model A) and finally we compared these with alternative definitions of AKI by incrementally adding criteria 4–6 to the model (models B, C and D). We calculated sensitivity by dividing the number of patients with biochemical and ICD-10 AKI by number of patients with ICD-10 AKI, with 95% confidence intervals. As ICD-10 includes only a subset of AKI, we did not feel it would be appropriate to report positive predictive values, specificity or a receiver operating characteristic curve.

To estimate the Grampian incidence of AKI based on NHS England algorithm criteria, we used the entire Grampian biochemistry dataset (*n* = 127,851) rather than those in the linked cohort (*n* = 49,718) and reported the number that met the AKI algorithm criteria in 2003. All analysis was conducted using Stata/SE 13.0 (StataCorp 2013).

## RESULTS

### Patient characteristics

For a cohort of 49 718 patients, there were 215 461 blood tests in 2003, 128 741 during hospital admissions and 86 720 from outpatients and the community. There were 4545 patients with 14 127 biochemical AKI alerts. Table 3 summarizes the characteristics of patients with abnormal and normal kidney function. In the abnormal eGFR group, biochemical AKI by NHS England criteria was present in 4373 (14.7%) patients and ICD-10 AKI in 329 (1.1%) patients. In the normal eGFR group, biochemical AKI was present in 172 (0.9%) patients and ICD-10 AKI in <5 patients. Patients with an abnormal eGFR were older and had more ischaemic heart disease, cardiac failure, diabetes or previous renal disease than patients with a normal eGFR (Table 3). The majority of index creatinine tests could be compared with a previous reference test within 1 year (92.1% abnormal eGFR group, 68.5% normal eGFR group). In 2003, the median number of tests per patient was two.

**Table 3.** Characteristics of the GLOMMS-II Cohort

Group	Abnormal eGFR in 2003	Normal eGFR in 2003 (20% sample)	Overall
*N*	29 729	19 989	49 718
Male sex (%)	10 994 (37.0)	9460 (47.3)	20 454 (41.1)
Age (IQR)	74 (66–81)	53 (39–66)	68 (54–78)
Age ≥ 70 (%)	19 590 (65.9)	3580 (17.9)	23 170 (46.6)
IHD (%)	4512 (15.2)	1019 (5.1)	5531 (11.1)
CCF (%)	1868 (6.3)	175 (0.9)	2043 (4.1)
Diabetes (%)	2121 (7.1)	397 (2.0)	2518 (5.1)
Renal disease (%)	1641 (5.5)	299 (1.5)	1940 (3.9)
ICD-10 AKI (%)	329 (1.1)	<5 (–)^a^	<334 (0.7)^a^
Biochemical AKI (%)	4373 (14.7)	172 (0.9)	4545 (9.5)
Criterion 1 (8–365 days) (%)	3277 (11.0%)	80 (0.4%)	3357 (6.8%)
Criterion 2 (1–7 days) (%)	1895 (6.4%)	101 (0.5%)	1996 (4.0%)
Criterion 3 (48 h) (%)	2218 (7.4%)	36 (0.2%)	2254 (4.5%)
Highest eGFR in 2003 (IQR)	57.2 (49.0–67.2)	84.6 (74.4–97.9)	70.0 (55.0–85.3)
Median no. of tests (IQR)	2 (1–6)	1 (1–2)	2 (1–4)
Reference test < 1 year (%)	152 308 (92.1)	29 550 (68.5)	181 858 (87.2)

Summary data as median and interquartile range (IQR) where appropriate; eGFR, estimated glomerular filtration rate; IHD, ischaemic heart disease; CCF, congestive cardiac failure.

^a^Suppressed information to prevent patient identification.

### Description of biochemical AKI using the NHS England algorithm

**FIGURE 2:**
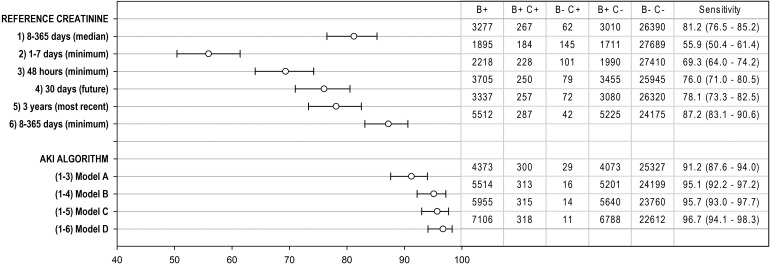
Sensitivity for ICD-10 AKI. Firstly of each of the individual AKI criteria (1–6) and then the combined criteria of the NHS AKI algorithm (model A) and modified algorithms (models B, C and D). Sensitivity with 95% confidence limits. B, Biochemical AKI; C, ICD-10 AKI. Note that combining criteria 2 and 3 detects 2748 patients with biochemical AKI, 244 with ICD-10 AKI and 85 without (sensitivity 74.2%, 69.1–78.8).

As few (<5) had ICD-10 AKI (reference standard) in the normal eGFR group, we report here only patients with and without biochemical AKI and ICD-10 AKI in the abnormal eGFR group (Table 4, Figure 2).

**Table 4.** The NHS AKI algorithm by peak AKI stage in patients with eGFR < 60 mL/min/1.73m^2^

Peak NHS AKI algorithm alert	AKI Stage 1	AKI Stage 2	AKI Stage 3	Any AKI	No AKI
*N*	2957	865	551	4373	25 356
Male sex (%)	1320 (44.6)	401 (46.4)	286 (51.9)	2007 (45.9)	8987 (35.4)
Age (IQR)	76 (67–83)	76 (65–83)	73 (62–81)	76 (66–83)	74 (66–81)
Age ≥ 70 (%)	2059 (69.6)	576 (66.6)	328 (59.5)	2963 (67.8)	16 627 (65.6)
IHD (%)	701 (23.7)	176 (20.3)	110 (20.0)	987 (22.6)	3525 (13.9)
CCF (%)	372 (12.6)	86 (10.0)	68 (12.3)	526 (12.0)	1342 (5.3)
Diabetes (%)	391 (13.2)	91 (10.5)	71 (12.9)	553 (12.7)	1568 (6.2)
Renal disease (%)	273 (9.2)	67 (7.7)	77 (14.0)	417 (9.5)	1224 (4.7)
ICD-10 AKI (%)	75 (2.5)	71 (8.2)	154 (27.9)	300 (6.9)	29 (0.1)
Reference eGFR (IQR)	65.4 (48.5–82.8))	71.4 (53.6–93.6)	70.6 (45.9–95.4)	67.0 (49.2–85.7)	–
Reference eGFR < 30 (%)	203 (6.9)	19 (2.2)	81 (14.7)	303 (6.9)	–
Alerts received (IQR)	1 (1–2)	3 (2–5)	5 (3–9)	2 (1–4)	–
Community at first AKI alert (%)	636 (21.5)	130 (15.0)	78 (14.2)	844 (19.3)	–
Inpatient at first AKI alert (%)	2321 (78.5)	735 (85.0)	473 (85.8)	3529 (80.7)	–
First alert >2 days after last test (%)	1511 (51.1)	517 (59.8)	339 (61.5)	2367 (54.1)	–
First alert within 2 days of last test (%)	1446 (48.9)	348 (40.2)	212 (38.5)	2006 (45.9)	–
First alert more than 7 days after last test (%)	1132 (38.3)	394 (45.5)	268 (48.6)	1794 (41.0)	–
First alert within 7 days of last test (%)	1825 (61.7)	471 (54.5)	283 (51.4)	2579 (59.0)	–

Summary data as median and interquartile range (IQR) where appropriate.

eGFR, estimated glomerular filtration rate; IHD, ischaemic heart disease; CCF, congestive cardiac failure; AKI, acute kidney injury.

ICD-10 AKI was present in more patients with biochemical AKI than without biochemical AKI (6.9 versus 0.1%). The majority were in hospital at first detection (80.7%) although only 45.9% had had a blood test in the previous 2 days. Patients with biochemical AKI had more previous ischaemic heart disease, cardiac failure, diabetes mellitus and renal disease than those without biochemical AKI.

**FIGURE 3:**
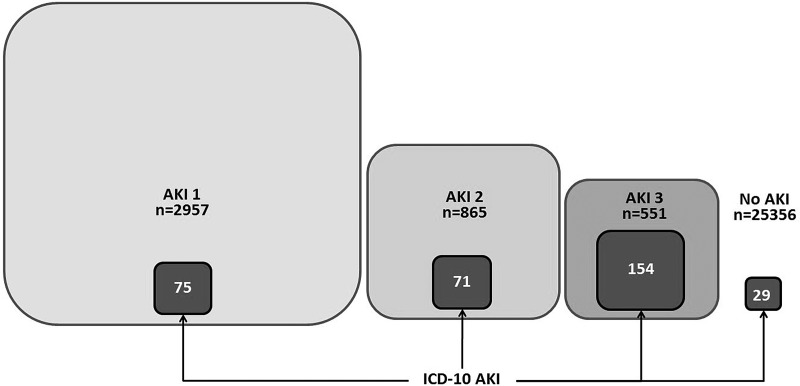
Number of patients at each stage by the NHS AKI algorithm and number with ICD-10 AKI. (Scaled representation). Includes 29 patients missed by the algorithm.

The peak AKI stage was Stage 1 for 2957, Stage 2 for 865 and Stage 3 for 551 patients. The median number of alerts increased over AKI Stages 1–3 (1, 3 and 5 alerts, respectively); as did the proportion of patients with ICD-10 AKI (2.5, 8.2 and 27.9%, respectively) (Figure 3). Of those with peak AKI of Stage 3, 264 (47.9%) had progressed from Stages 1 and 2 at initial detection.

Twenty-nine (8.8%) patients with ICD-10 AKI were missed by NHS England AKI criteria (Table 5). ICD-10 AKI patients missed were of similar age, sex and peak eGFR to those not missed, but had less ischaemic heart disease (13.8 versus 24.3%), cardiac failure (10.3 versus 15.3%) and diabetes (3.4 versus 15.7%) (Table 5).

**Table 5.** ICD-10 AKI patients detected and missed by the NHS AKI algorithm.

Patients with ICD-10 AKI	Detected by AKI algorithm	Missed by AKI algorithm
*N* (%)	300 (91.2)	29 (8.8)
Male (%)	155 (51.7)	15 (51.7)
Age (IQR)	75 (65–82)	77 (59–85)
Age ≥ 70 (%)	199 (66.3)	18 (62.1)
IHD (%)	73 (24.3)	4 (13.8)
CCF (%)	46 (15.3)	3 (10.3)
Diabetes (%)	47 (15.7)	1 (3.4)
Renal disease (%)	53 (17.7)	7 (24.1)
Highest eGFR in 2003 (IQR)	60.4 (39.9–87.2)	59.1 (34.8–77.0)

Summary data as median and interquartile range (IQR) where appropriate.

eGFR, estimated glomerular filtration rate; IHD, ischaemic heart disease; CCF, congestive cardiac failure; AKI, acute kidney injury.

### Biochemical AKI definitions versus ICD-10 AKI

Six different criteria (Table 2) for the reference value used to define biochemical AKI are evaluated in Figure 2. The number with AKI identified by each criterion ranged from 1895 to 5512. The proportion of ICD-10 AKI patients identified by each criterion (sensitivity) ranged from 55.9% (lowest creatinine 1–7 days) to 87.2% (lowest creatinine 8–365 days) (Figure 2).

NHS England AKI criteria, model A, defines AKI if one of criteria 1–3 is met (Table 2); 4373 patients had biochemical AKI by NHS England criteria and this included 300 of 329 patients with ICD-10 AKI (91.2% sensitivity). The algorithm missed 29 ICD-10 AKI patients (8.8%). The incremental addition of further criteria (models B, C and D) improved algorithm sensitivity. Adding a retrospective diagnosis (model B) reduced the number of patients missed to 16 (95.1% sensitive) and combining all criteria (model D) reduced this to 11 (96.7% sensitive), but with 7106 patients alerted (2733 more than model A).

### Population incidence of first episode of AKI

We also used NHS England algorithm criteria to estimate the incidence of AKI in unlinked biochemistry data for the entire adult Grampian population in the region in the same year (2003). There were 5565 patients with AKI out of an adult population of 438 332, of which 127 851 had blood tests. This equates to 12 696 adult patients per million population per year. In comparison, twice as many patients (10 811 patients, 24 664 per million population/year) met AKI model D criteria.

## DISCUSSION

In a large Scottish cohort, we assessed the sensitivity of the NHS England AKI biochemistry algorithm for an ICD-10 clinically confirmed subset of AKI patients. Using NHS England criteria, the 1-year risk of at least one AKI episode was estimated at 12 696 per million population and appropriately, ICD-10 AKI patients made up a greater proportion of patients with Stage 3 AKI (27.9%) than Stage 1 AKI (2.5%).

Encouragingly, NHS England algorithm criteria detected 91.2% of ICD-10 AKI. The 29 (8.8%) patients missed had similar age and kidney function (by peak eGFR) to those detected, but a lower proportion of diabetes and cardiac disease. Less frequent follow up in these patients may have resulted in fewer blood tests from which to diagnose AKI. The combination of criteria in the NHS England algorithm missed fewer ICD-10 AKI patients than any individual criterion.

Adding a retrospective diagnosis of AKI if serum creatinine subsequently fell captured half of the cases missed by NHS England criteria although this strategy would not be possible in real-time clinical practice. Further improvements in sensitivity could also be achieved by lengthening the look-back period or adding *lowest* rather than *median* creatinine criteria. An 8–365-day median criterion may have under-detected a minority of patients by providing a falsely high reference creatinine in some patients. Thus, using algorithm model D, only 11 cases were missed (3.3%). Each modification, however, resulted in substantially more patients alerted, up to 2-fold when the most sensitive model (D) was tested in the full Grampian population. This implies the presence of false positives.

Our assessment of the NHS England AKI algorithm has strengths in size and completeness covering an entire regional population, with availability of outpatient and private blood tests enabling us to use the AKI algorithm without the need for a hospital admission. A detailed comparison with other definitions also highlights the strengths of the algorithm. In the abnormal eGFR group, 92.1% of tests had a reference test within 1 year and 97.3% of these patients were linkable to an ICD-10 profile. The remaining 2.7% represent people without any record of hospital admissions (1999–2009). Exclusion of patients receiving chronic RRT prevented misclassification.

A difficulty among AKI studies is the absence of a perfect gold standard for assessing diagnostic accuracy. In previous attempts to define baseline kidney function, Siew *et al.* [15] tested a selection of baseline definitions using as reference standard an adjudicated value determined by two nephrologists. In their study of CKD and AKI patients with at least two previous tests within 24 months, the mean creatinine within 7–365 days provided best correlation [15]. In contrast, we found that the novel combination of NHS England algorithm criteria was more sensitive than any single definition using a clinically relevant reference standard.

The three combined criteria of the NHS England algorithm were also more sensitive for ICD-10 AKI than the two criteria used in a recent randomized controlled trial that did not report benefit from alerts [12]. Wilson and colleagues restricted their AKI criteria to changes within the previous week (criteria 2 and 3), but in our study we found this reduced sensitivity for ICD-10 AKI to 74.2%. In addition, at the time of first alert, we found that 41% of NHS England algorithm patients had a reference creatinine more than a week ago. These are patients who would either have received no alert or a delayed alert in the trial. A total of 67.3% of AKI occurs at the point of hospital admission or within two days of admission [29], but in the trial of Wilson and colleagues most alerts occurred more than two days after admission. Delays in alerting may therefore have reduced the potential for improving outcomes. This highlights the need to assess the timeliness of an alert algorithm as well as its accuracy.

As the NHS England algorithm is intended to improve AKI recognition, we focussed on assessing its sensitivity, using ICD-10 clinical coding (N17) as a subset of clinically confirmed AKI patients. N17 had a high specificity for the KDIGO AKI definition in a single centre in 2005 [23] and for this reason we also restricted to N17 codes (‘acute renal failure’), and not N19 (‘unspecified kidney failure’). ICD-10 AKI is therefore a useful reference test for testing sensitivity, but as it is only a subset of AKI we could not use it to assess specificity or false-positive rates and this is a limitation of our study. Nevertheless, the presence of false-positives is implied by the large variation in patients alerted with subsequent modifications to the algorithm. Further testing of specificity and positive predictive values would be appropriate, in addition to providing evidence of clinical benefit.

A further limitation is the time period (2003) as AKI has recently gained greater attention and coding has increased [23]. Nevertheless, while more patients are recognized and coded, the specificity of ICD-10 AKI remained unchanged between 2005 and 2010 [23]. As our study relied on a correct diagnosis in ICD-10 rather than high proportion of true AKI patients being coded, our use of codes in this time period is justified and our findings applicable.

We deviated from the NHS England algorithm in two respects. As sample time data were incomplete, we used ‘2 days’ rather than ‘48 h’ for criterion 3. This could overestimate AKI, so in sensitivity analysis we reduced the window to 1 day, which would underestimate AKI. With this change, 52 patients moved from biochemical AKI to no AKI on NHS England criteria, none with ICD-10 codes. In addition, we included only the highest creatinine on each day. This prevented spurious samples causing false positives, but could underestimate AKI. In sensitivity analysis all results were included and 127 patients without biochemical AKI now had AKI, including four with ICD-10 codes. These changes do not greatly affect our findings, but do reflect some of the considerations required for the pragmatic application of an automated algorithm without manual checking.

Notably, oliguria is not in the NHS England algorithm, but is part of the KDIGO AKI criteria and may be the first sign of AKI [6]. Some patients with ICD-10 AKI were missed due to inadequate baseline data and could be found by extending the look-back, but a lack of oliguria data may be another explanation.

Finally, in this study, we identified AKI patients rather than AKI events. This means that we did not capture repeat admissions, or changes in clinical care. While this has advantages in not over-representing AKI recurrence or misdiagnosing non-recovery as a repeat event, changes in clinical care such as hospital admission and transfers to critical-care setting should be investigated in future studies.

We noted three challenging groups of patients in this study. First, if a patient presents with an abnormal creatinine but no previous tests, AKI may only become apparent if the creatinine subsequently falls. The NHS England AKI algorithm includes a flag for such tests (13 064 blood tests in our cohort), but this requires good clinical judgment. Second, a patient may develop AKI but still have a normal eGFR and be overlooked. This was rare (AKI occurred in 0.9% of patients with normal tests), but these patients are still identified by the algorithm and should not be dismissed on absolute values. Third, 19.3% of AKI patients were out of hospital at first detection. Future studies should develop an evidence base on how an alert algorithm would affect the community.

Our key finding in this study was the encouraging performance of the NHS England algorithm (92.1% sensitive in detecting clinically ICD-10 AKI), but coded cases were still missed. There were also large variations in patients detected and missed with algorithm modifications. The timeliness of an alert may also be influenced by different definitions of AKI. The AKI definition is important not only in automated AKI detection in clinical practice where there is a trade-off between sensitivity and overburdening clinicians with ‘alert fatigue’ [13, 14], but also in clinical research where inconsistent definitions will result in selection biases. A distinction should be made between automated detection to assist early detection, and automated detection for diagnosis and clinical research. In research involving AKI patients, we recommend reporting the patient selection method so that limitations are transparent. In clinical practice, we show that automated alerts may be helpful, but the diagnosis of AKI depends on the clinician who must recognize cases of misclassified CKD or AKI without a previous baseline. Evidence of survival benefit is also still lacking. It is necessary to understand the best way to deliver alerts in a timely fashion so that they target the right patients and circumstances where most benefit can be provided.

In this study, the combined NHS England AKI algorithm criteria performed well as a diagnostic adjunct, identifying patients with an ICD-10 AKI code better than any individual AKI definition or previously studied definition. A trade-off existed between diagnostic sensitivity and ‘alert burden’. Clinicians should be mindful of this when interpreting an alert for an individual patient.

## FUNDING

This work was funded through a personal fellowship for S.S. supported by the Wellcome Trust (reference number 102729/Z/13/Z).

## CONFLICT OF INTEREST STATEMENT

S.S. is supported by a Clinical Research Training Fellowship from the Wellcome Trust. L.T. is funded by the Wellcome Trust as an Intermediate Clinical Fellow. C.B. is funded by, and a member of the Farr Institute @Scotland. S.S. and A.M. are also members of the Farr Institute @Scotland. N.F., G.P. and W.S. have no declarations. The results presented in this paper have not been published previously in whole or part, except in abstract format.

(See related article by Kellum *et al.* Can decision support systems work for acute kidney injury? *Nephrol Dial Transplant* 2015; 30: 1786–1789.)
